# An Optoelectronic Equivalent Narrowband Filter for High Resolution Optical Spectrum Analysis

**DOI:** 10.3390/s17020348

**Published:** 2017-02-10

**Authors:** Kunpeng Feng, Jiwen Cui, Hong Dang, Weidong Wu, Xun Sun, Xuelin Jiang, Jiubin Tan

**Affiliations:** 1 Center of Ultra-Precision Optoelectronic Instrument, Harbin Institute of Technology, Harbin 150080, China; aifenglin@gmail.com (K.F.); sired_hit@163.com (H.D.); weidongwuhit@foxmail.com (W.W.); sx2503962673@163.com (X.S.); jiangxuelin@gmail.com (X.J.); jbtan@hit.edu.cn (J.T.); 2 Shanghai Micro Electronics Equipment Co., Ltd., Shanghai 201203, China

**Keywords:** optical spectrum analysis, optical sensors, optical filters, coherent optics

## Abstract

To achieve a narrow bandwidth optical filter with a wide swept range for new generation optical spectrum analysis (OSA) of high performance optical sensors, an optoelectronic equivalent narrowband filter (OENF) was investigated and a swept optical filter with bandwidth of several MHz and sweep range of several tens of nanometers was built using electric filters and a sweep laser as local oscillator (LO). The principle of OENF is introduced and analysis of the OENF system is presented. Two electric filters are optimized to be RBW filters for high and medium spectral resolution applications. Both simulations and experiments are conducted to verify the OENF principle and the results show that the power uncertainty is less than 1.2% and the spectral resolution can reach 6 MHz. Then, a real-time wavelength calibration system consisting of a HCN gas cell and Fabry–Pérot etalon is proposed to guarantee a wavelength accuracy of ±0.4 pm in the C-band and to reduce the influence of phase noise and nonlinear velocity of the LO sweep. Finally, OSA experiments on actual spectra of various optical sensors are conducted using the OENF system. These experimental results indicate that OENF system has an excellent capacity for the analysis of fine spectrum structures.

## 1. Introduction

Optical filters are essential in a wide range of applications, including optical communications [1], spectroscopy [2,3], electronics [4] and optical sensors [5,6,7,8,9]. In particular, high resolution optical spectroscopy of high performance optical sensors has always attracted considerable interest and the characteristics of optical spectrum analysis (OSA) such as resolution, dynamic range and measurable range are highly dependent on optical filters. With the development of high resolution OSA, demands for optical filters with a narrowband ranging from GHz to several MHz and a wide swept range upwards to several ten nanometers are rapidly increasing. However, these optical filters cannot be realized through common technologies. 

To satisfy the requirements of new generation OSA of high performance optical sensors, much effort has been dedicated to the investigation of swept narrowband optical filters. For example, fiber Bragg gratings (FBGs) inscribed in polymer optical fiber (POF) or POF devices have considerable losses and weak spectra [5,6,7,8,9]. Conventional optical filter technologies available include dispersion gratings, arrayed waveguide gratings, thin-film dielectric interference filters, Fabry–Pérot (FP) filters, and Mach–Zehnder interferometers (MZI). However, these devices have difficulties in providing both narrowband and wide sweep ranges which hinders the development of the resolution and the measurable range of OSA technology. Several methods have been proposed to implement the desired optical filters. Resonance-based optical filter photonic circuits integrated on silicon-on-insulator have become an attractive technology because of its potential for miniaturization and easy tuning. Micro ring- [10] and Sagnac loop within a ring resonator-based optical filters [11] were proposed to build optical filters with bandwidth in the GHz range and swept ranges of several tens of nanometers. Nevertheless, their bandwidth is proportional to swept range. This means their fine range is fixed and cannot be infinite. Next, researchers utilized non-linear optics principles, including stimulated Brillouin scattering (SBS), stimulated Rayleigh scattering (SRS) and four-wave mixing (FWM) to construct optical filters [3,4,12]. Narrowband optical filters based on SBS in optical fibers were first proposed as a result of their highest gain among optical fibers. The gain spectrum bandwidth of SBS is several MHz and its gain spectrum can be swept with a tunable laser. This technique achieved a spectral resolution of 80 fm (~10 MHz) and a sweep range of 40 nm in an 11.5-km length dispersion-shifted fiber [2]. Later, superposition of three Brillouin lines and vector attributes of SBS amplification in standard, weakly birefringent fibers, were utilized to improve its spectral resolution to 3 MHz [12]. To reduce the bandwidth, FWM was investigated and Brillouin dynamic grating with a narrowband of 2.4 MHz in a 100-m length single-mode fiber (SMF) was reported [13]. Subsequently, Dong et al. optimized the SMF length to achieve an approximate birefringence-free SMF section between 0 m and 300 m and minimum spectral resolution as low as 0.5 MHz for optical spectrometry was demonstrated [3]. For the SBS and FWM with SBS non-linear optics principles, the bandwidth of their optical filters cannot be further reduced because the intrinsic Brillouin bandwidth is several MHz in range. Preussler and Schneider combined the SBS polarization pulling effect and heterodyne detection technique and achieved a resolution in the attometer or lower kilohertz range at 1550 nm [14]. Recently, Zhu et al. used non-uniform fiber or trapped fiber to increase the SBS threshold value 7 dB higher than that of the conventional SMF and the higher SBS gain spectrum was well suppressed [4,15]. Thus, a SRS spectrum bandwidth of less than 10 kHz in 27-km length SMF was achieved and optical filters based on SRS were utilized in an ultra-narrowband fiber laser. Nevertheless, how to realize a swept SRS filter for OSA technology is still an open issue. On the other hand, all optical filters based on non-linear optics principles need several hundred meters or even several tens of kilometers of SMF, which makes them highly sensitive to environmental factors, such as temperature drifts and vibrations. The weak nonlinear optics effects lead to a low spectral gain or a weak refractive index modulation and the dynamic range of these technologies are not adequate. 

In this paper, an optoelectronic equivalent narrowband filter (OENF) for high resolution OSA of high performance optical sensors is investigated and a swept optical filter with a bandwidth of several MHz and swept range of several tens of nanometers is built using electric filters and a sweeping laser as local oscillator (LO). The OENF is based on the coherent optics principle. The homodyne and heterodyne components between a signal under test (SUT) and the LO are processed by an electric filter, thus only the frequency components within the electric filter can achieve the following processes. The bandwidth of electric filters can be narrow and frequency components within the electric filter represent the optical power spectral density (OPSD) of SUT around the wavelength of LO, so a swept narrowband filter can be achieved when the LO sweeps past the SUT. The bandwidth of OENF can be adjusted through the design of electric filters and its swept range is determined by the LO. This technology avoids using long SMF and overcomes the contradiction between narrowband and wide range. 

This paper is organized as follows: Section 2 demonstrates the OENF principle and establishes influence of OENF on OSA results. Section 3 describes our experimental setup of an OENF system and verifies its principle through experiments. Finally, conclusions are presented. 

## 2. Principle 

### 2.1. Principle of OENF

**Figure 1:**
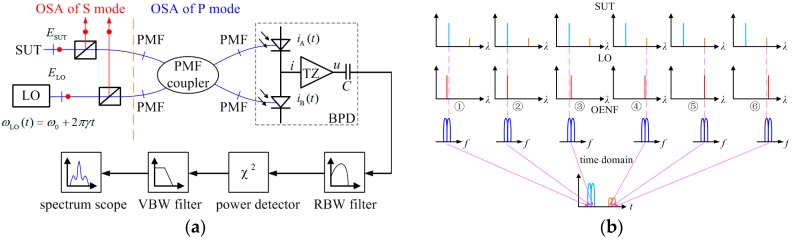
(**a**) Schematic diagram of the OENF system; (**b**) Working process of the OENF system.

A schematic diagram of an OENF system is illustrated in Figure 1a. The P polarized mode and S polarized mode of the SUT whose spectrum is to be characterized are split by a polarizing beam splitter (PBS) to respectively perform OSA which benefits from the coherent optics principle of OENF. The P polarized modes of LO and SUT are combined in a polarization-maintaining fiber (PMF) coupler to generate two wideband coherent optical signals. A balanced photodetector (BPD) is utilized to receive coherent optical signals. A transimpedance amplifier (TZ) and blocking capacitor C within the BPD extract the heterodyne component and convert its photocurrent into composite alternating-current (AC) voltage signals. A composite AC voltage signals whose frequency is located in the narrowband resolution bandwidth (RBW) filter could pass the RBW filter. Then signals directly characterizing the OPSD of SUT at the LO wavelength are obtained and subsequently measured using a power detector to get the root-mean-square (RMS). When a swept LO sweeps at a linear rate, RMS describing OPSD of SUT at the wavelength of swept LO is continuously exported by the power detector. Figure 1b shows this process. RBW filter filtering of wideband coherent signals can be equivalent to an OENF whose center is located at the LO wavelength. The transfer function of the RBW filter in the frequency domain and envelope function of OENF in the time domain keep the same shape when the LO linearly sweeps and the bandwidth of the RBW filter is wide enough to contain a few tens of periods of coherent signal [16,17]. Figure 1b also indicates the LO wavelength and corresponding OENF of six points in the time domain during a sweep. Subsequently, the relationship between the RMS or envelope and the swept LO in the time domain is achieved as shown at the bottom of Figure 1b. Then, the RMS or envelope is smoothed by a video bandwidth (VBW) filter and digitized. Finally, the SUT spectrum is shown on the scope. For OSA of FBGs inscribed in polymer optical fiber and POF devices having considerable losses and weak spectra [5,6,7,8,9], an OENF system has remarkable benefits compared with commercial OSA: (1) much more fine special structures of a broad FBG such as chirped FBG in POF can be scoped, which may be related to the special sensing characters, manufacture inspection and other applications; (2) weak spectra can be analyzed as a result of the fact that the OENF system is based on coherent theory. 

The signal process of OENF system has already been established in [16,17,18,19,20]. The LO sweeps past the SUT and forms a chirp-coherent signal in both the frequency and time domains. The chirp coherent signal characterized as a linear-frequency-modulation chirp signal in the time domain and a transfer function of the RBW filter the in frequency domain can thus be transformed into a filter envelope function in the time domain as an OENF for OSA. OENF has significant influence on the OSA results, as discussed in the next subsection. 

### 2.2. Analysis of the Influence of OENF on OSA Results

An arbitrary SUT spectrum can be assumed as a combination of monochromatic components [21,22]. OSA using OENF can be implemented through the analysis of every monochromatic signal. The monochromatic SUT is utilized to evaluate the influence of OENF on the OSA results, including power uncertainty and spectral resolution, and then SUT is extended to be an ensemble of complex monochromatic components to complete the analysis. 

The coherent signal between the monochromatic SUT and sweep LO is processed by a RBW filter and the output of this RBW filter in the time domain can be achieved. Then, a RMS power detector detects chirp signals within RBW filter and the signal representing its RMS power $\sqrt{\frac{1}{T}\int_{0}^{T}y^{2}\left(t\right)dt}$ is continuously exported to form the time-domain RMS envelope of the amplitude product of swept OENF and monochromatic SUT on the scope. The OPSD $P_{SUT}$ of SUT is proportional to the integration of the square of RMS envelope function S(t). Based on Parseval’s theorem, the OPSD $P_{SUT}$ of SUT can be also written in a form of frequency domain as:

(1) $$P_{SUT}=\frac{1}{2\pi }{\int_{-\infty }^{+\infty }\left|{\tilde{u}}_{AC}(\omega )\right|}^{2}{\left|H(\omega )\right|}^{2}d\omega =\int_{0}^{t}S^{2}(t)dt$$

where, ${\tilde{u}}_{AC}(\omega )$ is frequency-domain signal of swept OENF, H(ω) is the transfer function of RBW filter and S(t) is the time-domain RMS envelope function of the swept OENF. 

**Figure 2:**
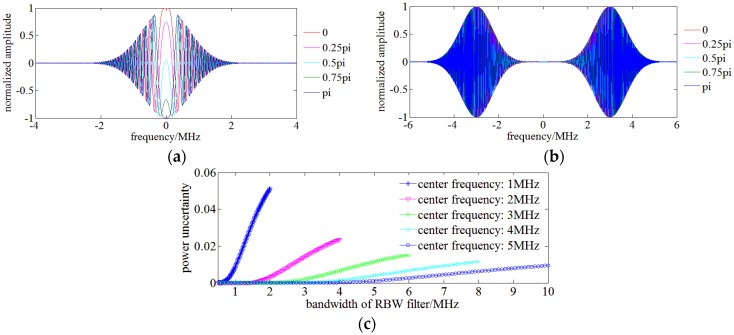
(**a**) Process of chirp signal with different initial phase using a low pass RBW filter; (**b**) Chirp signal process with different initial phases using a band pass RBW filter; (**c**) The relationship between power uncertainty and the parameters of the RBW filter.

However, the coherent signal between the monochromatic SUT and sweep LO relates to their initial phase difference Δφ which determines the chirp signal within the RBW filter. A low pass Gauss filter with a bandwidth of 1 MHz and a bandpass Gauss filter with a center frequency of 3 MHz and a bandwidth of 1 MHz is for example employed as RBW filter. The chirp signals within RBW filters in frequency domain are illustrated in Figure 2a,b. Based on Equation (1), Figure 2a,b explicitly indicate that the phase difference Δφ changes the power of the frequency-domain chirp signal within the bandwidth or pass band of the RBW filter and its influence on a low pass RBW filter is more significant. 

The ratio of maximum OPSD error to minimum OPSD by varying the phase oscillating component of integrand kernel function represents the OPSD accuracy of SUT:

(2) $$e_{P_{SUT}}=\frac{\max P_{SUT}(\Delta \varphi )-\min P_{SUT}(\Delta \varphi )}{\min P_{SUT}(\Delta \varphi )}$$

Figure 2c shows the relationship between power uncertainty and parameters of a Gauss RBW filter. RBW filters allowing more low frequency components to pass are more easily influenced by the phase difference Δφ and have a much higher power uncertainty, which agrees with the analysis based on Figure 2a,b, so a low pass RBW filter is not adopted for OSA and a narrow bandwidth and high center frequency RBW filter help reduce the power fluctuation. On the other hand, the BPD DC blocking capacitor also prevents low frequency components from entering the RBW filter and subsequent processing modules. 

**Figure 3:**
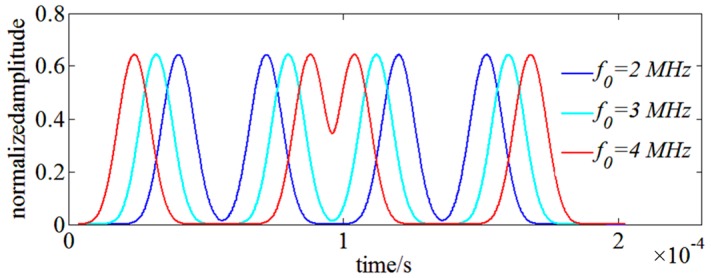
The relationship between overlapping of double monochromatic signals with the center frequency $f_{0}$ of RBW filter.

The bandwidth and the center frequency of RBW filter also influence the spectral resolution of OENF for OSA. The bandwidth Δf of RBW filter is set to be 1 MHz and simulations are run by varying its center frequency $f_{0}$ from 2 MHz to 4 MHz. SUT consists of two monochromatic signals of 1550 nm and 1550 nm + 10 MHz. Swept LO starts at 1550 nm − 7 MHz with a sweep velocity of 1 nm/s. Figure 3 shows the simulation result and it indicates that a sufficiently high center frequency leads to overlapping between double monochromatic signals which limits the spectral resolution. The spectral resolution of OENF for OSA can be an simply estimated as $2f_{0}+\Delta f$. Considering the power uncertainty results of Figure 2c, a highest spectral resolution of 5 MHz with acceptable low power uncertainty of monochromatic SUT can be achieved with $f_{0}=2\;\mathrm{MHz}$ and Δf=1 MHz.

Once the parameters of the RBW filter for the highest spectral resolution are determined, the type of the RBW filter becomes an issue. The engineered realizable filters include the following types: Butterworth, Chebyshev, Gauss and Bessel. Butterworth, Chebyshev filter and Gauss is chosen as RBW filters but the Bessel filter is not considered due to its worse amplitude-frequency characteristics. The absolute value of the transfer function of Butterworth filter can be expressed as:

(3) $$H(\omega )=\frac{1}{\sqrt{1+{\left(\frac{\omega -\omega_{0}}{\Delta \omega /2}\right)}^{2n}}}$$

where *n* is the order of the Butterworth filter, $\omega_{0}$ is the center angular frequency of the filter and Δω is the 3 dB bandwidth of the filter. 

The absolute value of the transfer function of Chebyshev filter can be expressed as:

(4) $$H(\omega )=\frac{1}{\sqrt{1+\xi^{2}T_{n}^{2}\left(\frac{\omega -\omega_{0}}{\Delta \omega }\right)}}$$

where ξ is the ripple factor, $T_{n}$ is *n*th order Chebyshev polynomial. 

The absolute value of the transfer function of Gauss filter can be expressed as:

(5) $$H(\omega )=\exp\left(-\ln\sqrt{2}\frac{{\left(\omega -\omega_{0}\right)}^{2}}{{\left(\Delta \omega /2\right)}^{2}}\right)$$

**Figure 4:**
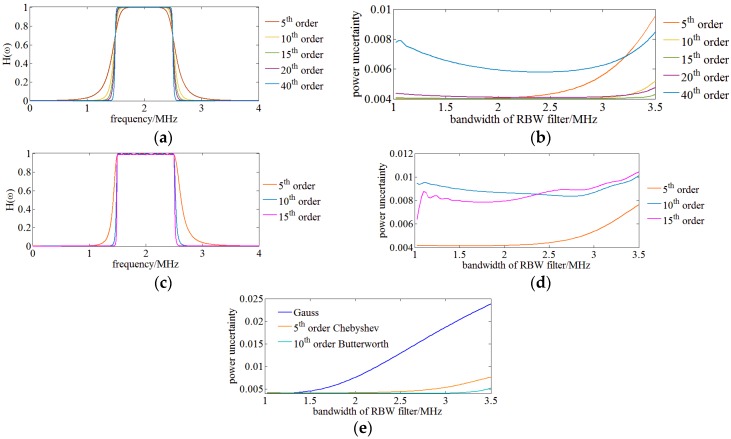
(**a**) The absolute value of the transfer function of Butterworth filters of different orders; (**b**) Power uncertainty of Butterworth filters of different orders as RBW filters; (**c**) The absolute value of the transfer function of Chebyshev filters of different orders; (**d**) Power uncertainty of Chebyshev filters of different orders as RBW filters; (**e**) Comparison of power uncertainty among Gauss, 5th order Chebyshev, 10th order Butterworth RBW filters.

The influence of RBW filters of different types and orders on the power uncertainty of OSA results is investigated. Figure 4a,c show the absolute value of the transfer function of Butterworth and Chebyshev filters of different orders. Simulations are run with these RBW filters of OENF for OSA and their power uncertainties are illustrated in Figure 4b,d. Simulations indicate that the power uncertainty of Butterworth filters initially decreases with the raise of order but rapidly increases when the order of the Butterworth filters reaches 40th and the power uncertainty shows its local minimum at a medium bandwidth of the RBW filter, which means that there is a power uncertainty balance between the benefit of increased bandwidth and the shortcoming of allowing low frequency components to pass. On the other hand, the power uncertainty of Chebyshev filters increases directly with the raise of order. Both the Butterworth and Chebyshev filters of high orders are close to an ideal band pass filter as a rectangle shape in the frequency domain decreasing the power uncertainty, which means a filter having smooth and fast attenuation characteristics could reduce the power uncertainty. Considering the physical realizability, a 10th order Butterworth and a 5th order Chebyshev filter were chosen for following analysis. 

Figure 4e shows the relationship between power uncertainty and bandwidth of different RBW filters, 10th order Butterworth, 5th order Chebyshev filter and a Gauss filter. It indicates that a 10th Butterworth achieves a minimum power uncertainty. 

**Figure 5:**
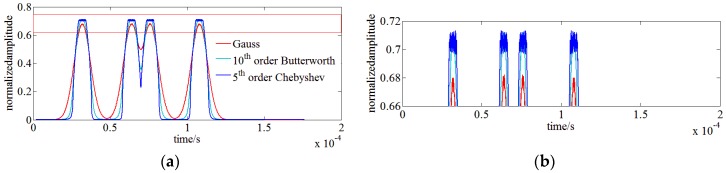
(**a**) Full view of the influence of RBW filters of different types on the spectral resolution; (**b**) Partial view of (a).

Besides power uncertainty, RBW filters of different types also have an influence on the spectral resolution of the OSA results. A simulation is run with the following configuration: SUT is modeled as two adjacent monochromatic signals with a frequency difference of 5.5 MHz; three filters of 10th order Butterworth, 5th order Chebyshev filter and Gauss filter having a same center frequency of 2 MHz and bandwidth of 1 MHz are respectively employed as RBW filters of the OENF. Figure 5 shows the time domain envelopes of the monochromatic SUT of the three RBW filters. It indicates that the Gauss filter has the largest overlap between double monochromatic signals, leading to the worst spectral resolution and the Chebyshev filter has the biggest ripples in the pass band which has an influence on power uncertainty. Therefore, the 10th order Butterworth filter is appropriately utilized as RBW in our OENF system. 

**Figure 6:**
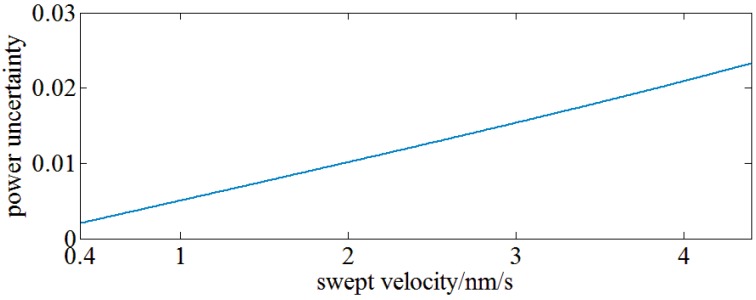
The relationship between power uncertainty and swept velocity of swept LO.

On the other hand, the period number of chirp signals within the RBW filter varies with the swept velocity of the sweep LO which is also a factor leading to power uncertainty. Actually, the period number is inversely proportional to the sweeping velocity, so a chirp signal within the RBW filter with a high swept velocity is sparse and more sensitive to the phase diversity. Figure 6 shows the relationship between power uncertainty and sweep velocity with a 10th order Butterworth RBW filter of 2 MHz center frequency and 1 MHz bandwidth. A very short measurement time may be achieved by rising the sweep velocity of a swept LO at the cost of power uncertainty. 

**Figure 7:**
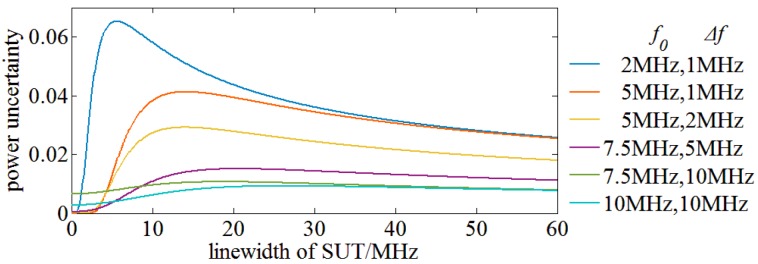
The power uncertainty of complex monochromatic SUT achieved with Butterworth RBW filter of different center frequency and bandwidth.

Finally, SUT is modeled as an ensemble of complex monochromatic components having a certain linewidth. The process of evaluating the power uncertainty is similar to that of a monochromatic SUT, but the power uncertainty terms of monochromatic signals experience a double integration to obtain the power uncertainty of complex monochromatic SUT. Figure 7 shows the power uncertainty of complex monochromatic SUT achieved with RBW filters of different center frequency and bandwidth. It indicates that a RBW filter with a higher center frequency could reduce the power uncertainty; a wider bandwidth of RBW filter could reduce the power uncertainty of a wide linewidth of SUT at the cost of spectral resolution. However, a decrease of spectral resolution has no influence on the OSA results of wider SUT with less fine spectral structures. The power uncertainty decreases rapidly with the center frequency $f_{0}$ of RBW filter increasing from 2 MHz to 7.5 MHz, but the change is slowed down when the center frequency is higher than 7.5 MHz. On the other hand, the power uncertainty is further reduced through broadening of the pass band Δf, so two 10th order Butterworth filters of $f_{0}=7.5\;\mathrm{MHz}$, Δf=10 MHz and $f_{0}=10\;\mathrm{MHz}$, Δf=10 MHz are chosen as candidate RBW filters. Considering the spectral resolution, a 10th order Butterworth RBW filter of 7.5 MHz center frequency and 10 MHz bandwidth is finally utilized in our 0.2 pm medium spectral resolution OENF system for OSA. 

## 3. Experiments and Discussion

### 3.1. Experimental Setups

The experimental setup of the OENF system is as same as the schematic diagram illustrated in Figure 1. The swept LO is an external cavity tunable (ECT) laser with a linewidth of 100 kHz and a fiber laser with a linewidth of 1 kHz. The all-fiber PBS and PMF coupler are made by the AFR Laser Company (Zhuhai, Guangdong, China) and the working axes are aligned to the slow axes of PMF. The PDB is switchable gain and bandwidth PDB450C produced by Thorlabs (Newton, NJ, USA) with coverage of 800‒1700 nm. An AC form of PDB450C is utilized and it has a common mode rejection ratio of over 30 dB. The power detector is an AD8361 (Analog Devices, Inc., Norwood, MA, USA) which is used for true power measurement and has a working bandwidth up to 2.5 GHZ with a high linear response. The integral time of the AD8361 can be customized. RBW filters are designed using a software named FilterPro (Analog Devices, Inc., Norwood, MA, USA) and the circuits are built with an AD4817 with a high gain bandwidth product of 1 GHz which can ensure the actual transfer function is as least distortionless as possible. The VBW filters are also designed using FilterPro and their circuits are made of OP37 (Analog Devices, Inc., Norwood, MA, USA) with a medium gain bandwidth product of 63 MHz. The analog signals are digitalized by a PCI-1714 produced by ADVANTECH (Taipei, Taiwan) with a maximum sampling rate of 10 MHz. Software for acquiring and processing data from the PCI-1714, and displaying SUT spectra is embedded on computer. 

### 3.2. Verification of the OENF’s Principle

In this section, simulation and experiment results are compared to verify the OENF’s principle. 

**Figure 8:**
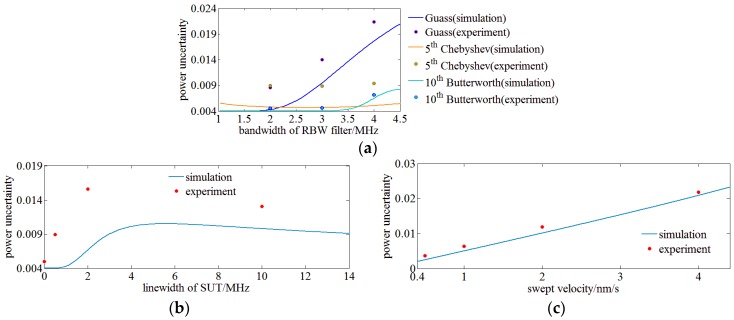
(**a**) Verification of RBW filter’s type on power uncertainty of OENF system for OSA; (**b**) Verification of SUT’s linewidth on power uncertainty of OENF system for OSA; (**c**) Verification of swept velocity of LO on power uncertainty of OENF system for OSA.

First, the influence of RBW filter type on the power uncertainty of OENF system for OSA is experimentally demonstrated. SUT is a fiber laser with a linewidth of 1 kHz. The wavelength of the fiber laser as SUT is 1550 nm and the sweep LO is an ECT laser sweeping in the range of 1550 ± 0.25 nm. Gauss, 5th order Chebyshev and 10th order Butterworth RBW filters with a center frequency of 2 MHz varies their bandwidth. These configurations are used to run the simulation and conduct experiments. The swept LO sweeps 30 times and the spectrum power is analyzed by the OENF system. The maximum and minimum power are achieved and the power uncertainty is calculated using Equation (2). Figure 8a shows the comparison of power uncertainties by experiment with simulation results. It indicates that simulation results are consistent with the experimental data and errors may come from the noise and drift of the circuits. The error between simulation and experiment of the 10th order Chebyshev filter is larger than that of the other filters which may be caused by ripples in its pass band. Figure 8a also verifies that the 10th order Butterworth RBW filter has a minimum power uncertainty according to the experimental data. 

Second, the influence of the SUT’s linewidth on the power uncertainty is verified. SUT of 1 kHz, 500 kHz, 2 MHz and 10 MHz and a sweeping fiber laser of 1 kHz as LO are utilized to conduct experiments and run simulations. A 10th order Butterworth filter with a center frequency of 2 MHz and a bandwidth of 1 MHz is employed as RBW filter. The results are illustrated in Figure 8b and have a similar trend, but the error increases rapidly with the SUT’s linewidth which may be caused by the phase noise and intensity noise of SUT of a wide linewidth. 

**Figure 9:**
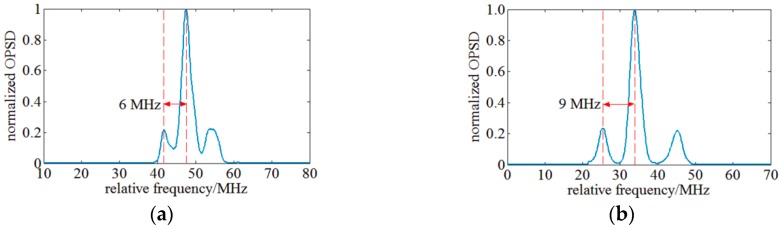
(**a**) OSA of 6 MHz frequency interval spectrum signal; (**b**) OSA of 9 MHz frequency interval spectrum signal.

Third, the influence of sweep velocity of the LO on the power uncertainty is investigated. Base on the analysis of Section 2, the sweep velocity of the sweep LO changes the period number of chirp signals within the bandwidth of the RBW filter and makes the OENF system more sensitive to phase diversity. SUT of this experiment is a fiber laser with a linewidth of 1 kHz at 1550 nm and the swept LO is an ECT laser sweeping in the range of 1550 ± 0.25 nm. A 10th order Butterworth filter a center frequency of 2 MHz and of a bandwidth 1 MHz is employed as RBW filter. Figure 9a shows the relationship between sweep velocity and power uncertainty. It indicates that both simulation and experiment results agree with each other and the power uncertainty increases with the sweep velocity. 

Finally, the spectral resolution capacity is experimentally tested. An intensity modulated fiber laser of 1 kHz linewidth is employed as SUT for OSA. According to Jacobi-Anger identity, the fiber laser modulated by LiNbO_3_ modulator has several peaks and their frequency interval is equal to the modulation frequency. The modulation signal is configured to achieve maximum 1st order peaks and zero 2nd order peaks. A 10th order Butterworth filter with a center frequency of 2 MHz and a bandwidth of 1 MHz is used as RBW filter and a 25 kHz low pass filter is used as VBW filter within the OENF system. Results are illustrated in Figure 9 and they indicate that a modulation signal of 6 MHz frequency interval (~48 fm in 1550 nm wavelength range) can be distinguished, so a swept OENF can achieve a spectral resolution of 6 MHz. 

The experimental spectral resolution is slightly worse than the designed value which may be caused by the difference between the realized engineering and the theoretically achievable RBW filter. During the 6 MHz spectral resolution test, the resolution of the OENF system reaches its limit and the broader peak on the right may be caused by insufficient spectral resolution. The bandwidth of sidebands of the 9 MHz spectral resolution test is nearly equal. The frequency distance of both sidebands is not equal which is caused by the non-linear sweep velocity of the LO. The wavelength calibration system could calibrate the sweep velocity every 1 pm, however, the sweep velocity within 1 pm is unknown and this induces chirp. 

### 3.3. Real-Time Wavelength Calibration of Swept LO

During the OSA, the LO sweeps the spectrum of SUT at a nominal velocity and the PCI-1714 acquires the analog signals with its inner clock at an equal time interval. However, the swept LO has phase noise and nonlinear velocity. These factors have deeply influences on the results of OSA, specifically, the spectrum shape. The wavelength of the swept LO should be calibrated in real time, so that the sampling points of data acquisition card (DAQ) can be connected with the wavelength. 

**Figure 10:**
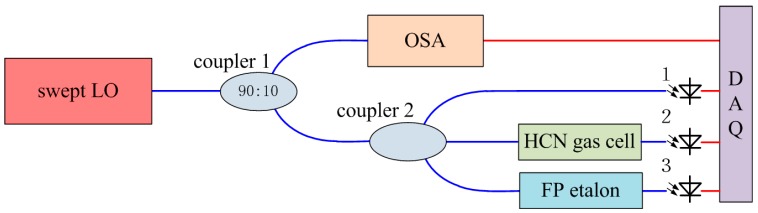
Schematic diagram of the RTWC system.

The real-time wavelength calibration (RTWC) system is illustrated in Figure 10. The swept LO is divided into two parts at a ratio of 90:10, one part is for OSA and the other is for RTWC. Then the part of the LO sweep entering the RTWC system is equally divided into three parts by a 1 × 3 fiber coupler, one part passes a hydrogen cyanide (HCN) gas cell for absolute wavelength calibration, one part passes a FP etalon, and another is directly detected by a photoelectricity conversion device for power normalization [23]. 

The HCN gas cell has specific absorption lines covering from 1530 nm to 1560 nm in the C-band. The HCN gas cell is made by Wavelength References (Corvallis, OR, USA) and it is a NIST-traceable H^13^C^14^N gas cell at 100 Torr (NIST SRMs 2519). The wavelength of specific absorption lines has an expanded uncertainty of less than ±0.2 pm. The FP etalon is made by Micron Optics (Atlanta, GA, USA) and it has a nominal free spectral range (FSR) of 0.8 pm. The transmission peaks of FP etalon are very close in wavelength. This can be used to determine the real-time wavelength of the sweep LO and sampling dates between two transmission peaks can be linearly interpolated to determine the real-time wavelength. 

**Figure 11:**
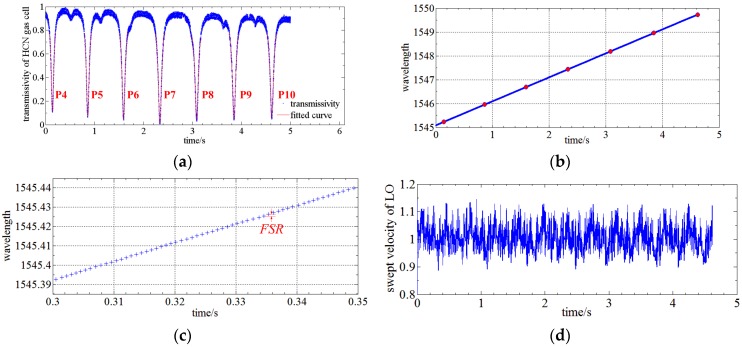
(**a**) Signal processing of HCN; (**b**) Signal processing of FP; (**c**) Detailed view of (**b**); (**d**) Sweep velocity of LO at nominal velocity of 1 nm/s.

Figure 11 shows the signal processing of the RTWC system. The signals of the HCN gas cell and FP etalon are first normalized to suppress the LO power drift. Then, the absorption lines of HCN gas cell is fitted using a Voigt profile [24]. The pseudo-Voigt profile is utilized in this paper for approximation of the Voigt profile using a linear combination of a Gaussian curve and a Lorentzian curve instead of their convolution. The pseudo-Voigt profile can be written as:

(6) $$V\left(\lambda \right)=\eta \exp\left[-\frac{4\ln2{\left(\lambda -\lambda_{0}\right)}^{2}}{\gamma^{2}}\right]+\left(1-\eta \right)\frac{\gamma^{2}}{{\left(\lambda -\lambda_{0}\right)}^{2}+\gamma^{2}}$$

where, $\lambda_{0}$ is the center wavelength, γ is the bandwidth of a Gaussian curve and a Lorentzian curve, η is the ratio of Gaussian curve and a Lorentzian curve. 

The initial wavelength of the sweep LO is roughly known, which means that the initial wavelength is certainly located between two certain absorption lines. The absorption lines during the sweeping period are determined once the direction of the LO sweep is known using Equation (6). The upper illustration of Figure 11a shows that the absorption lines are successively P4 (1545.23033(7) nm), P5 (1545.95549(7) nm), P6 (1546.69055(8) nm), P7 (1547.43558(24) nm), P8 (1548.19057(7) nm), P9 (1548.95555(4) nm) and P10 (1549.73051(4) nm). The center of FP etalon is located using the centroid method and the bottom illustration of Figure 11a shows the location of the transmission peaks of the FP etalon versus sampling time. Calculating the number of transmission peaks of the FP etalon between two absorption lines of HCN gas cell, the average FSR of 0.7998 pm can be achieved, so the DAQ sampling points can be connected with the wavelength in real-time and a wavelength accuracy of ± 0.4 pm can be achieved during OSA. 

Using this RTWC system and signal processing method, the LO sweep velocity at a nominal velocity of 1 nm/s is calibrated as shown in Figure 11d. The sweep velocity is about ±10% around 1 nm/s. Therefore, the influence of phase noise and nonlinear velocity of the LO sweep can be effectively reduced in this way. 

### 3.4. OSA of Fine Spectrum Structures of Optical Sensors Using the OENF System 

In this section, optical spectra of optical sensors lighted by both wide amplified spontaneous emission (ASE) sources and narrow laser sources are measured using the proposed OENF system and these results are compared with the results obtained by an Anritsu OSA (Anritsu, Kanagawa, Japan) of 30 pm spectral resolution. 

First, the ASE spectrum of a phase-shift fiber Bragg grating (PS-FBG) sensor is measured. The spectrum of the PS-FBG sensor is comparatively wide and the bandwidth can reach ~0.3 nm, however, it has a very narrow notch of ~1 pm in the reflectance spectrum. A 10th order Butterworth filter with a center frequency of 7.5 MHz and a bandwidth of 10 MHz is used as RBW filter and a 5 kHz low pass filter is used as VBW filter of the OENF system. The theoretical spectral resolution is 25 MHz (~0.2 pm in 1550 nm wavelength range). LO is an ECT laser. 

**Figure 12:**
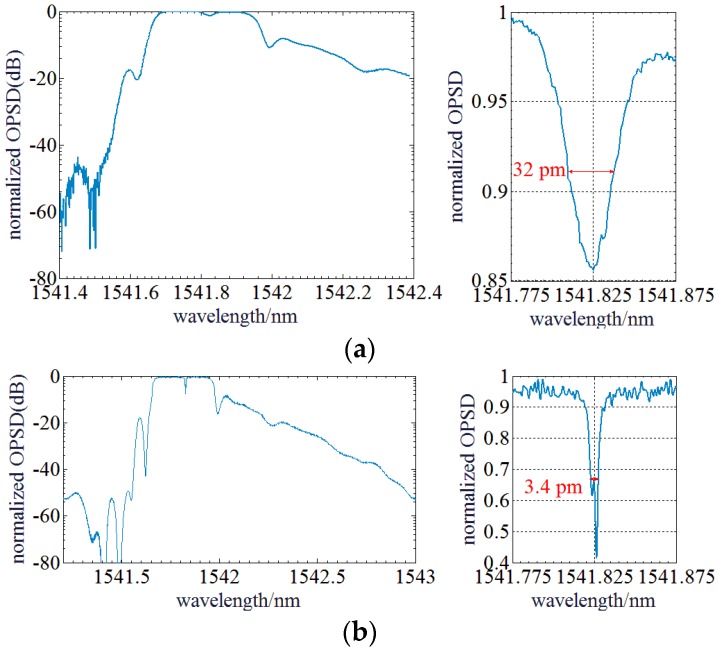
(**a**) Optical spectrum of the PS-FBG sensor measured by an Anritsu OSA. (**b**) Optical spectrum of the PS-FBG sensor measured by the OENF system.

Figure 12 shows the reflectance spectrum and spectral notch structure of PS-FBG sensor measured by both the Anritsu OSA and the OENF system. It indicates that the reflectance spectrum profiles are the same but the result obtained by the OENF system has some ripples which are probably caused by the intensity noise of the ASE source. However, the measured spectral notch structures of the PS-FBG sensor are quite different. The OENF system gives a much narrow spectral notch which has more spectrum fine information and it is much closer to the real spectrum, but the result obtained by the Anritsu OSA only provides a ~32 pm notch, which is the actual spectral resolution of this OSA and any fine information within the narrow notch is lost. 

**Figure 13:**
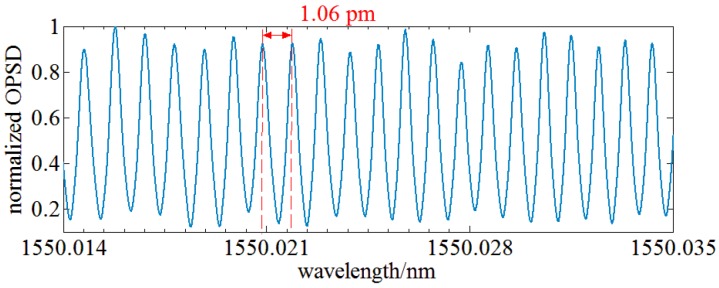
Optical spectrum of a fiber MZI sensor measured by the OENF system.

Second, the optical spectrum of a fiber MZI sensor is measured using the OENF system. It has a sinusoidal spectrum with a norm spectral period of 1 pm ± 10%. An ASE source serves as input of the fiber MZI sensor and a sinusoidal spectrum can be achieved using this fiber MZI sensor. A 10th order Butterworth filter with a center frequency of 7.5 MHz and a bandwidth of 10 MHz is used as RBW filter and a 5 kHz lowpass filter is used as VBW filter of the OENF system. LO is an ECT laser. Figure 13 shows the optical spectrum measured by the OENF system. The optical spectrum has a sine shape and the optical spectral period is 1.06 pm within the uncertainty of the norm value of the fiber MZI sensor. 

**Figure 14:**
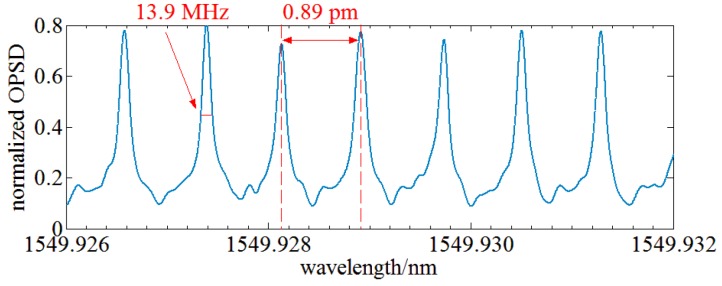
Optical spectrum of an optical frequency comb measured by OENF system.

Finally, the optical spectrum of an optical frequency comb with a norm comb tooth spacing of 0.88 pm is tested using the OENF system. A 10th order Butterworth filter with a center frequency of 2 MHz and a bandwidth of 1 MHz is used as RBW filter and a 25 kHz lowpass filter is used as VBW filter of the OENF system. The LO is an ECT laser. The result is illustrated in Figure 14. 

The achieved comb tooth spacing is 0.89 pm and the linewidth of each comb is 13.9 MHz. The measurement error is less than 1.5%. Combs of an optical frequency comb inevitably have power diversity, so the measured OPSD diversity of each comb may result from the optical frequency comb source.

## 4. Conclusions

In this paper, an OENF technology for OSA is theoretically and experimentally investigated. The principle of OENF is briefly introduced and much effort is paid to analyzing the influence of OENF on OSA. RBW filters are found to have a significant influence on the spectral resolution and power accuracy, two key parameters of an OENF system. Considering the spectral resolution, a RBW filter of 2 MHz center frequency and 1 MHz bandwidth can achieve an appropriate power uncertainty for monochromatic SUT. The power uncertainty can be further reduced by decreasing the sweep velocity. Subsequently, three types of engineering-realizable RBW filters: Gauss, Chebyshev and Butterworth of different orders are respectively simulated to evaluate their power uncertainty and spectral resolution. A 10th order Butterworth RBW filter of 2 MHz center frequency and 1 MHz bandwidth has a smallest power uncertainty and a better spectral resolution than a 5th order Chebyshev RBW filter. This 10th order Butterworth filter is thus chosen as the RBW filter for high spectral resolution applications. In a similar way, a 10th order Butterworth RBW filter of 7.5 MHz center frequency and 10 MHz bandwidth is employed as RBW for medium spectral resolution applications. In order to reduce the influence of the phase noise and nonlinear velocity of the LO sweep, a RTWC system consisting of a HCN gas cell and FP etalon is proposed to determine the absolute and relative RTWC. With the help of the RTWC system, the wavelength accuracy of OSA can be raised to ±0.4 pm in the C-band and the shape distortion of the spectrum is reduced. Next, the OENF principle is verified by experiments. The results show that the experimental power uncertainty results agree with those of the simulation; the maximum power uncertainty is less than 2%, and a spectral resolution of 6 MHz can be achieved by OENF system. Finally, experiments on OSA of actual spectra including a PS-FBG sensor, fiber MZI sensor and an optical frequency comb using OENF system are conducted. Experimental results indicate that OENF system could achieve more spectrum fine information than possible with a commercial Anritsu OSA. 

It can be concluded that the proposed OENF technology can simultaneously achieve a narrow bandwidth and a wide sweep range. The OENF system can also resist the drift and vibration and thus work at industrial sites. These properties could lead to a widely applicability in the fields of optical communication, spectroscopy, electronics, optical sensors and photography.
